# Beclin-1 expression is retained in high-grade serous ovarian cancer yet is not essential for autophagy induction *in vitro*

**DOI:** 10.1186/s13048-015-0182-y

**Published:** 2015-08-04

**Authors:** Rohann J. M. Correa, Yudith Ramos Valdes, Trevor G. Shepherd, Gabriel E. DiMattia

**Affiliations:** Translational Ovarian Cancer Research Program, London Regional Cancer Program, London, Ontario Canada; Department of Biochemistry, Schulich School of Medicine & Dentistry, The University of Western Ontario, London, Ontario Canada; Department of Obstetrics & Gynaecology, Schulich School of Medicine & Dentistry, The University of Western Ontario, London, Ontario Canada; Department of Oncology, Schulich School of Medicine & Dentistry, The University of Western Ontario, London, Ontario Canada; Department of Anatomy & Cell Biology, Schulich School of Medicine & Dentistry, The University of Western Ontario, London, Ontario Canada; London Regional Cancer Program, 790 Commissioners Road East, Room A4-919A, London, Ontario Canada N6A 4 L6

**Keywords:** High-grade serous ovarian cancer, Spheroid, Autophagy, Beclin-1

## Abstract

**Background:**

Autophagy is a conserved cellular self-digestion mechanism that can either suppress or promote cancer in a context-dependent manner. In ovarian cancer, prevalent mono-allelic deletion of *BECN1* (a canonical autophagy-inducer) suggests that autophagy is impaired to promote carcinogenesis and that Beclin-1 is a haploinsufficient tumor suppressor. Nonetheless, autophagy is known to be readily inducible in ovarian cancer cells. We sought to clarify whether Beclin-1 expression is in fact disrupted in ovarian cancer and whether this impacts autophagy regulation.

**Methods:**

*BECN1* expression levels were assessed using The Cancer Genome Atlas (TCGA) datasets from 398 ovarian high-grade serous cystadenocarcinomas (HGSC) and protein immunoblot data from HGSC samples obtained at our institution. Knockdown of *BECN1* and other autophagy-related gene expression was achieved using siRNA in established human ovarian cancer cell lines (CaOV3, OVCAR8, SKOV3, and HeyA8) and a novel early-passage, ascites-derived cell line (iOvCa147-E2). LC3 immunoblot, autophagic flux assays, transmission electron microscopy and fluorescence microscopy were used to assess autophagy.

**Results:**

We observed prevalent mono-allelic *BECN1* gene deletion (76 %) in TCGA tumors, yet demonstrate for the first time that Beclin-1 protein expression remains relatively unaltered in these and additional samples generated at our institution. Surprisingly, efficient siRNA-mediated Beclin-1 knockdown did not attenuate autophagy induction, whereas knockdown of other autophagy-related genes blocked the process. Beclin-1 knockdown instead decreased cell viability without inducing apoptosis.

**Conclusions:**

Taken together, these data demonstrate that despite its sustained expression, Beclin-1 is dispensable for autophagy induction in ovarian tumor cells *in vitro,* yet may be retained to promote cell viability by a mechanism independent of autophagy or apoptosis regulation. Overall, this work makes novel observations about tumor expression of Beclin-1 and challenges the accepted understanding of its role in regulating autophagy in ovarian cancer.

**Electronic supplementary material:**

The online version of this article (doi:10.1186/s13048-015-0182-y) contains supplementary material, which is available to authorized users.

## Background

Macroautophagy (autophagy) is a conserved mechanism for the sequestration of cytoplasmic contents in membrane-bound vesicles and their subsequent lysosome-mediated degradation. It facilitates turnover of damaged organelles, removal of misfolded/aggregated proteins, and destruction of intracellular pathogens, ridding the cell of harmful components [1] and liberating constituent biomolecules to fuel metabolic and biosynthetic pathways [2]. In this way, autophagy plays an essential role in maintaining cellular homeostasis.

Autophagy is negatively regulated by growth-factor signaling through the PI3K/AKT pathway – specifically by the downstream activity of mechanistic target of Rapamycin complex 1 (mTORC1). If mTORC1 activity is suppressed, however, an autophagy-inducing complex is allowed to form and convey activating signals to downstream effectors [3]. These effectors are mammalian homologues of autophagy-related (“*atg”*) proteins originally discovered in yeast [4]. They play pivotal roles in the initiation of autophagic membrane formation, deployment of these membranes to envelop cytoplasmic contents, and the delivery of this cargo to lysosomes for degradation [5].

Among the *ATG*s, *BECN1* (mammalian homologue of yeast *atg6* that encodes Beclin-1) was one of the earliest discovered and has been extensively studied. It functions in a core complex with Class III PI3K (PI3K C3) [6] and p150 [7] as a canonical initiator of autophagy [8]. Mice harboring heterozygous disruption of the *Becn1* gene (*Becn1*^+/−^) are viable but develop malignancies at higher rates than wild-type littermates [9]. Moreover, the human chromosomal *BECN1* locus (17q21) exhibits single-copy loss in prostate [10] and breast cancers [11, 12]. In ovarian cancer, heterozygous loss is most prevalent, affecting up to 70 % of tumors [13–17]. Therefore, Beclin-1 – and autophagy by extension – are thought to be tumor suppressive.

Although homeostatic autophagy in normal tissues may initially curtail tumorigenesis, evidence exists for autophagy upregulation in established tumors [18]. This may serve as an adaptive response to mitigate cellular stresses that typify tumor pathobiology, including intrinsic stresses such as high metabolic demands [19] and ER-stress [20] as well as extrinsic stresses such as anti-neoplastic agents [21] and the tumor microenvironment itself [e.g., hypoxia [22], reduced nutrient [23] and growth factor availability [24]]. In ovarian cancer, autophagy induction was classically demonstrated by Lu *et al.* in 2008 [25] and in numerous other studies since then, including our own work [26]. Most recently, increased autophagy in recurrent tumor nodules on the peritoneal surface relative to patient-matched primary ovarian tumors has been described, suggesting that autophagy is important the setting of ovarian cancer metastasis [27].

Since ovarian tumors appear capable of undergoing autophagy despite prevalent *BECN1* heterozygous loss, we wondered if Beclin-1 was actually downregulated in this context and whether it was still required for autophagy induction. Here we demonstrate that even with prevalent *BECN1* single-copy loss, Beclin-1 protein expression remains similar across 398 high-grade serous ovarian tumors. Yet surprisingly, knockdown of Beclin-1 had no effect on autophagy induced by either pharmacologic or non-pharmacologic stimuli. It did, however, reduce cell viability in an apoptosis-independent manner in two cell lines tested. Therefore, Beclin-1 appears non-essential for autophagy induction in ovarian cancer *in vitro* cultures. Nonetheless, its sustained expression may contribute to cell viability by a currently undefined mechanism.

## Materials & methods

### Isolation of tumor cells from patient tissues

All work with patient materials has been approved by the Western University Health Sciences Research Ethics Board (Protocol numbers 12668E and 16391E). The majority of samples were collected from patients with stage II-IV high-grade serous ovarian carcinoma (Additional file 1: Table S1). Ascitic fluid collected at time of paracentesis or debulking surgery was used to generate primary ascites cell cultures as described previously [28]. Solid tumor tissue from metastatic lesions was obtained at time of debulking surgery. Briefly, tissue was dissected into cubes ~2-5 mm^2^ in size, wrapped in aluminum foil, snap-frozen on dry ice, and stored at −80 °C. To generate lysates, samples removed from −80 °C were mixed with dry ice pellets and pulverized using a mortar and pestle. The powdered sample was then added to lysis buffer and lysates prepared as previously described [26].

### Culture of ovarian cancer cell lines

Human ovarian cancer cell lines were purchased from the American Type Culture Collection (ATCC; Manassas, VA) and cultured in Dulbecco's modified Eagle's medium (SKOV3, CaOV3) or RPMI-1640 (OVCAR8, HeyA8) supplemented with 5 % fetal bovine serum (FBS; Wisent). Early-passage cell lines (designated “iOvCa”) are derived from ascites cultures (designated “EOC”) of the corresponding number (e.g., iOvCa201 is a line derived from EOC201). Early-passage lines were maintained in DMEM/F12 medium (Gibco/Invitrogen, Carlsbad, CA) supplemented with 10 % FBS. The iOvCa147-E2 line is a clone of iOvCa147. To establish stable expression of eGFP-LC3B, sub-confluent (~60-70 %) OVCAR8 cells were transfected with the pBMN-ires-puro-eGFP-LC3B construct (gift of Dr. C. McCormick, Dalhousie University) and transferred to Puromycin-containing selection medium (1 μg/mL) where clones with robust eGFP-LC3B expression were isolated. All cells were maintained in a 37 °C humidified atmosphere of 95 % air and 5 % CO_2_. Adherent cells were maintained on tissue culture-treated polystyrene (Sarstedt, Newton, NC) and non-adherent cells were maintained on Ultra-low Attachment (ULA) cultureware (Corning, Corning, NY).

### Immunofluorescence and immunoblotting

Spheroids present in ascitic fluid at time of collection were isolated by filtration through a 40 μm cell-strainer (Becton Dickinson, Franklin Lakes, NJ), gently rinsed off the membrane with PBS into a collection tube, embedded in cryo-matrix (Fisher, Ottawa, ON), and frozen on dry ice for storage at −80 °C. Frozen blocks were sectioned on a Shandon cryostat at 6 μm, mounted on slides, and stored at −20 °C until needed. Spheroid sections were processed, stained, microscopy performed, and images captured as was done previously for adherent cells on coverslips [26]. Anti-LC3 antibody (1:250; Cell Signaling Technology, Danvers, MA) was used to stain endogenous autophagosome-associated fluorescent puncta.

For immunoblotting, all samples (cultured cells or tumor tissue) were lysed in a supplemented RIPA buffer prepared as previously described [26]. Protein concentration was determined using Bradford’s method, immunoblots performed, chemiluminescent images captured, and band intensities quantified all as previously described [26].

### Antibodies and other reagents

Antibodies against pAkt-Ser473 (#9271), AKT (#9272), p-p70S6K (#9234), p70S6K (#2708), LC3 (#2775), Beclin-1 (#3738), Atg5 (#2855), and Atg7 (D12B11; #8558) were obtained from Cell Signaling Technology (Danvers, MA). Anti-actin antibody (A 2066) was purchased from Sigma. Akt inhibitor VIII (Akti-1/2) was purchased from EMD/Calbiochem (#12408; SanDiego, CA) and Chloroquine was purchased from Sigma (C-6628).

### Transmission electron microscopy

Adherent cells (80 % confluent) or spheroids (24 h following seeding to non-adherent culture) were collected, fixed, dehydrated, embedded in resin, sectioned, stained, and examined using transmission electron microscopy as previously described [26].

### Acquisition of The Cancer Genome Atlas (TCGA), Cancer Cell Line Encyclopedia (CCLE), and The Cancer Proteome Atlas (TCPA) datasets

Copy-number data for ovarian serous cystadenocarcinoma samples were generated from array comparative genomic hybridization data acquired at the Broad TCGA genome characterization center using the Affymetrix Genome-Wide Human SNP Array 6.0 platform. Raw data were analyzed using the GISTIC2 method to generate gene-level copy-number calls and downloaded from Memorial Sloan-Kettering Cancer Center’s cBioPortal for Cancer Genomics (http://www.cbioportal.org/) [29]. GISTIC2-generated copy-number estimates (log_2_-transformed values, not thresholded) were downloaded from the UCSC Cancer Browser (https://genome-cancer.ucsc.edu). Copy-number data for Cancer Cell Line Encyclopedia (CCLE) ovarian cancer lines were also generated using GISTIC2 and downloaded as copy number calls from cBioPortal.

Protein expression data was generated at the MD Anderson Cancer Center (MDACC) TCGA proteome characterization center using reverse-phase protein array (RPPA) technology and were normalized to sample medians as previously described [30]. RPPA data were downloaded as *z*-scores from the cBioPortal and as log_2_-normalized values from the UCSC Cancer Browser. RPPA datasets for CCLE ovarian cancer lines (batch 35) were obtained from MDACC’s TCPA website (http://app1.bioinformatics.mdanderson.org/tcpa/_design/basic/index.html).

Copy-number and RPPA datasets were last downloaded on July 3, 2014.

### siRNA transfection

For RNAi-mediated knockdown of gene expression, we utilized Dharmacon siGENOME SMARTpool reagents [Non-Targeting Control Pool #2 (D-001206-14-05), *BECN1* (M-010552-01), *ATG5* (M-004374-04), and *ATG7* (M-020112-01); Thermo Scientific, Waltham, MA]. Transfections were conducted according to manufacturer’s instructions with modifications described previously [26]. Importantly, double-transfection was used as described by Smith *et al.*, to maximize efficiency and duration of knockdown [31]. iOvCa147-E2 cells were seeded to 6-well plates at a density of 350,000/well whereas the density for CaOV3, OVCAR8, SKOV3, and HeyA8 cells were 200,000/well. Cells were counted and seeded for further experimentation 96 h following initial transfection.

### Assays of cell viability and apoptosis

*Adherent culture*: 96 h post-transfection, adherent cells were trypsinized and single-cell suspensions generated for cell counting. To enumerate viable cells, Trypan Blue reagent (Gibco/Invitrogen, Carlsbad, CA) was applied (1:1 dilution) and cells counted in a using a TC-10 automated cell counter (BioRad, Mississauga, ON) with two counts per replicate of experimental triplicates.

*Spheroid culture*: 96 h post-transfection, cells were trypsinized, counted and seeded to 24-well ultra-low attachment plates at a density of 5.0x10^4^ per well to form spheroids as previously described. Spheroids were collected, pelleted, and briefly loosened/disaggregated by trypsinization (~5 min) 72 h post-seeding. CellTiter-Glo reagent (Promega, Madison, WI) was prepared according to manufacturer’s instructions and added to spheroids in trypsin (1:1 volume ratio). The mixture was transferred to a white-walled 96-well microplate and luminescence detected in triplicate wells using a spectrophotometer (Wallac 1420 Victor 2; Perkin-Elmer, Waltham, MA).

To assess apoptosis, the Caspase-Glo 3/7 assay (Promega, Madison, WI) was used as per manufacturer’s instructions (1:1 volume ratio). Reagent was added directly to 96-well tissue culture plates containing adherent cells. Spheroids were first collected, centrifuged, and media aspirated until 100 μL remained and then equal volume of reagent added. Following 1.5 h incubation, the mixture was transferred to a white-walled 96-well microplate and luminescence measured using a microplate spectrophotometer (Wallac 1420 Victor 2; Perkin-Elmer, Waltham, MA). Measurements were made in triplicate wells. Additionally, a Human Apoptosis Antibody Array (R&D Systems, Minneapolis, MN) was used to screen for alteration in protein expression of 35 apoptosis-associated genes. Adherent and spheroid cells were collected and lysed at 24 h post-seeding using the supplied buffer, protein concentration quantified using Bradford’s method, and samples treated as per manufacturer’s instructions. Chemiluminescent images were captured and signal intensity quantified as previously described [26].

### Graphing and statistical analysis

All graphs were generated using GraphPad Prism 5 (GraphPad Software, San Diego, CA). Data were expressed as Mean ± SD or Mean ± SEM, as indicated. All statistical analyses [Student’s *t*-test, Analysis of Variance (ANOVA) with Newman-Keuls correction for multiple comparisons, as well as correlation and linear regression] were performed using GraphPad Prism 5. Tests of significance were set at *p* < 0.05.

## Results

### Despite prevalent single-copy loss of the BECN1 gene, Beclin-1 protein expression is maintained in high-grade serous ovarian tumors and ascites-derived cells

Heterozygous loss of the canonical autophagy gene *BECN1* is well documented in epithelial ovarian cancer cells [13–17]. To our knowledge, however, no study has yet determined whether hemizygosity at the *BECN1* locus results in correspondingly decreased expression at the protein level in ovarian tumors.

**Fig. 1:**
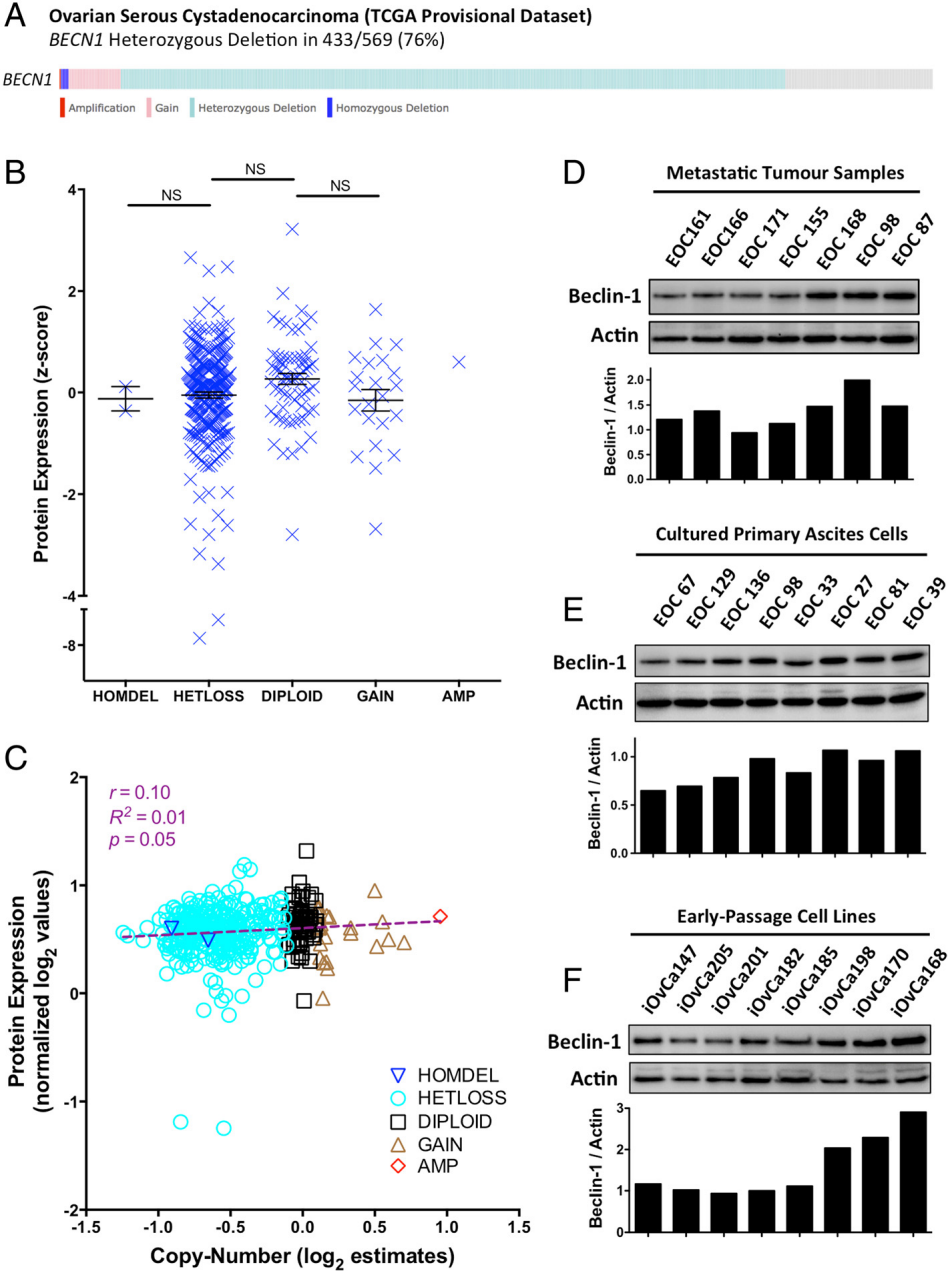
Beclin-1 protein expression is maintained in high-grade serous ovarian tumors and ascites-derived cells. **a** Gene copy-number calls at the *BECN1* locus are depicted for 569 ovarian serous cystadenocarcinoma tumors (teal & blue = heterozygous & homozygous deletion, respectively; red & pink = high-level & low-level amplification, respectively). OncoPrint generated using cBioPortal.org. **b** *BECN1* protein expression data (RPPA) expressed as z-scores and GISTIC2-based copy-number calls for 398 samples were downloaded from cBioPortal and plotted. One-way ANOVA with Tukey’s Test was performed (NS: p > 0.05). Lines: Mean ± SEM. **c** Log_2_-transformed and normalized RPPA data and GISTIC2-based copy-number estimates (log_2_-transformed) for 398 samples were downloaded from UCSC’s Cancer Browser and plotted. **d-f** Beclin-1 expression was quantified in fresh-frozen samples of metastatic tumor tissue (**d**), primary cultures of ascites-derived cells (**e**), and early-passage ascites-derived cell lines (**f**). Quantification was performed using densitometry and depicted relative to Actin

In order to assess the relationship between *BECN1* copy-number and expression, we interrogated level 3 array comparative genomic hybridization (aCGH) and RPPA data generated by TCGA from a large number of high-grade serous ovarian tumors (91 % of which are from metastatic, stage III-IV disease; Additional file 2: Table S2). Based on aCGH data that was processed to yield gene-level copy-number calls, 433/569 ovarian tumours (76 %) possessed hemizygous deletion of the *BECN1* gene (Fig. 1a). This confirms earlier reports of single-copy loss in ~70 % of tumors [10, 14–16].

To determine whether *BECN1* copy-number correlated with protein expression, we first plotted RPPA *z*-scores from 398 tumors against their copy-number calls. Interestingly, only 9 tumors with hemizygous *BECN1* loss had Beclin-1 expression lower than 2 standard deviations from the mean (*z*-score of −2; Fig. 1b). Moreover, we did not find a significant reduction in Beclin-1 protein abundance associated with decreased copy-number (*p* > 0.05). We also plotted normalized RPPA values against log_2_-transformed copy-number. Again, largely equivocal Beclin-1 expression was observed in samples regardless of gene copy-number (Fig. 1c), however linear regression and correlation analyses did reveal a weak (*r* = 0.10; *R*^*2*^ = 0.01) yet significant (*p* = 0.0455) correlation between copy-number and protein expression. Nonetheless, these data clearly demonstrate that in most tumors harboring single-allele loss of *BECN1*, its protein product is still expressed at approximately diploid levels.

Furthermore, metastatic tumor samples (Fig. 1d), primary ascites cell cultures (Fig. 1e), and early-passage cell lines generated at our institute (Fig. 1f) exhibited a similar trend in Beclin-1 protein expression. Samples identified as the highest- and lowest-expressers were assessed on the same immunoblot, demonstrating only modest differences in protein abundance analogous to the narrow range of expression observed in TCGA samples (Fig. 1c).

Collectively, these data show that the level of Beclin-1 protein in epithelial ovarian cancer specimens falls within an easily detectable narrow range regardless of copy number.

### Autophagy is inducible in ascites cells derived from patients with high-grade serous ovarian cancer (HGSC)

We previously demonstrated that ascites-derived cells cultured as adherent monolayers or multicellular aggregates (spheroids) induced cyto-protective autophagy in response to AKT inhibition, a well-known stimulus for autophagy induction [26]. Closer examination of these data led us to suspect that spheroid culture itself might serve as a sufficient stimulus for autophagy.

**Fig. 2:**
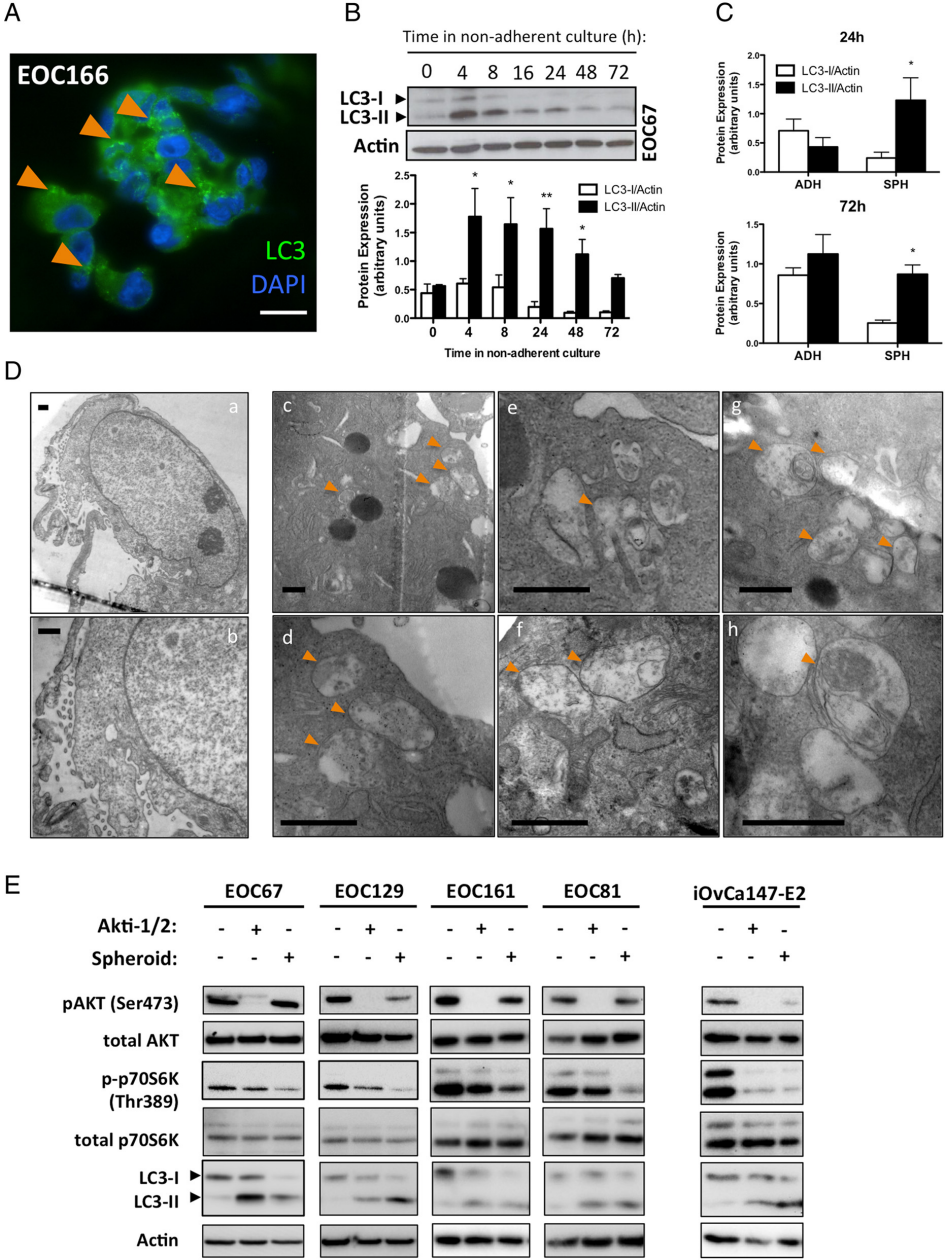
Autophagy is readily inducible in ascites-derived cells. **a** Spheroids filtered from patient ascites fluid for immunofluorescence analysis (n = 3 EOC samples). Nuclei (blue) and cytoplasmic LC3 staining (green) are visible. Orange arrowheads indicate LC3 puncta. Scale Bar: 20 μm. **b** EOC cells were seeded to non-adherent plates and lysates obtained at indicated time points. Immunoblot of EOC67 (top) and quantification of LC3-I and LC3-II expression relative to Actin (n = 3 EOC samples) were performed. Bars: Mean ± SEM; *p < 0.05, **p < 0.01. **c**. Quantifications also performed on immunoblots of parallel adherent (ADH) and (SPH) cultures of multiple EOC samples (n = 10) at 24 h (top) and 72 h (bottom). Bars: Mean ± SEM; *p < 0.05. **d** Parallel adherent (~80 % confluent) and spheroid cultures were analyzed by transmission electron microscopy. Orange arrowheads denote autolysosomes (autophagosomes that have fused with lysosomes). Scale bars: 500 nm. **e** Adherent (ADH) cultures (~80 % confluent) of the indicated samples were subjected to 24 h of Akti-1/2 treatment (EOCs: 5 μM; iOvCa147-E2: 3.5 μM) and protein lysates generated. Parallel spheroid cultures (24 h under non-adherent conditions) of the indicated samples were also simultaneously lysed and immunoblot performed for indicated proteins

To investigate this possibility, ascites fluid was collected from consenting patients with HGSC. These samples contain tumor cells in suspension that exist either as single cells or spheroids, the latter being considered an integral part of ovarian cancer metastasis (reviewed in [32]). Frozen sections of isolated spheroids were generated and immunofluorescence staining performed for microtubule-associated light-chain 3 (LC3), the most established marker of autophagy. This revealed LC3 protein localization to discrete puncta throughout the cytoplasm of spheroid-associated cells (Fig. 2a), consistent with the presence of autophagic vesicles or autophagosomes.

To further characterize spheroid-associated autophagy, ascites cell cultures (EOCs) were established from multiple patients, then transferred to non-adherent cultureware to generate *in vitro* spheroids as previously described [33]. Protein lysates of these cultures were generated for the indicated time-points and immunoblots performed for LC3, since its cleaved and lipidated form (LC3-II) is identifiable as a differentially migrating species that indicates the presence of autophagosomes. We observed a significant increase in abundance of LC3-II as early as 4 h post-seeding (*p* < 0.01), with the ratio of LC3-II:LC3-I greatest by approximately 24 h (once aggregates/spheroids have formed; Fig. 2b). This increased LC3-II:LC3-I ratio was maintained through 72 h of non-adherent culture despite a decrease in the absolute amounts of these species over that time, a phenomenon known to occur with extended autophagy (Fig. 2b). In addition to time-course analysis, we also assessed paired adherent/spheroid samples generated from ten EOC cultures, verifying significantly increased LC3-II relative to LC3-I at both the 24 h and 72 h time points (*p* < 0.05; Fig. 2c).

To verify that autophagy was proceeding through the degradation phase in spheroids (i.e., lysosomes fusing with autophagosomes), they were subjected to transmission electron microscopy (TEM). Ultra-thin sections of fixed spheroid cells were examined for autophagolysosomes (APLs): cytoplasmic vesicles bounded by double-membranes whose contents are undergoing degradation. APLs were not observed in adherent cultures (Fig. 2d, a-b), but were frequently noted in spheroids (Fig. 2d, c-h), suggesting that autophagy is induced and proceeds to the degradation phase in spheroid cells.

Finally, we compared spheroid-associated autophagy to pharmacologically-induced autophagy (treatment with Akti-1/2, an allosteric AKT inhibitor) that we demonstrated in our prior report [26]. Inhibition of AKT activity reduces mTORC1 activity allowing induction of autophagy. As evidenced by LC3 processing, spheroid-associated autophagy was comparable to that induced by Akti-1/2 in parallel adherent cultures (Fig. 2e). This was observed in primary ascites cell cultures (EOCs) as well as the early-passage cell line iOvCa147-E2 (derived from an ascites culture). Therefore, ascites-derived cells easily induce autophagy by both pharmacologic as well as spheroid-associated, non-pharmacologic means.

### Autophagy is induced in established ovarian cancer cell lines irrespective of endogenous Beclin-1 expression level or gene copy number

**Fig. 3:**
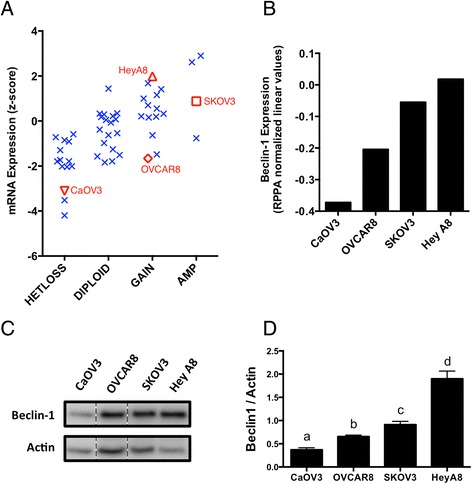
In established ovarian cancer cell lines, *BECN1* copy-number correlates with mRNA and protein expression. **a** Log2-transformed mRNA expression data for 52 ovarian cancer cell lines [Cancer Cell Line Encyclopedia (CCLE) datasets] are displayed as a function of *BECN1* copy number calls. Cell lines representative of low (CaOV3), intermediate (OVCAR8), and high (SKOV3, Hey A8) Beclin-1 expression were selected for further analysis. **b** Beclin-1 protein expression is depicted for the selected cell lines [normalized linear Reverse-Phase Protein Array (RPPA) values from The Cancer Proteome Atlas (TCPA) datasets]. **c** Independent protein lysates from adherent cultures (n = 3) were run on a single gel with representative lanes depicted for each cell line (intervening lanes cropped). **d** Beclin-1 expression was quantified relative to Actin. Bars: Mean ± SEM. One-way ANOVA with Newman-Keuls correction for multiple comparisons was performed. Letters denote statistically significant differences (n = 3 lanes per cell line, *p* < 0.05)

To further investigate the contribution of Beclin-1 expression to autophagy induction in ovarian cancer, representative lines with low, intermediate, and high Beclin-1 expression were selected based on datasets from the Cancer Cell Line Encyclopedia (CCLE) project [34]. *BECN1* copy-number and mRNA expression level were plotted for 51 ovarian cancer cell lines (Fig. 3a). From this, commonly-used lines were chosen for further analysis based on *BECN1* gene copy-number (SKOV3, amplification; HeyA8 and OVCAR8, low-level gain; CaOV3, heterozygous loss) and mRNA expression (CaOV3, low; OVCAR8, intermediate; SKOV3 and HeyA8, high). Quantitative protein expression data for these cell lines (obtained from The Cancer Proteome Atlas, TCPA) was in agreement with mRNA expression data (Fig. 3b). Likewise, in our hands, these four lines each expressed significantly different levels of Beclin-1 protein (Fig. 3c, d).

Despite differences in Beclin-1 expression and gene copy-number, all four lines were able to induce autophagy. In adherent cells, 24 h treatment with Akti-1/2 resulted in enhanced processing of LC3 protein and an increased abundance of LC3-II relative to untreated cells (Additional file 3: Figure S1A). The addition of a Chloroquine (CQ; 50 μM) pulse in the final 4 h preceding lysis resulted in a further increase in LC3-II in all three lines as expected given that CQ will stall autophagosome processing leading to a further increase in the LC3-II isoform (Additional file 3: Figure S1A), thus confirming autophagic flux. In non-adherent spheroids, the addition of a CQ pulse also increased LC3-II levels, likewise implying flux of spheroid-associated autophagy (Additional file 3: Figure S1B). Similar results were obtained in the early-passage, ascites-derived line iOvCa147-E2 (Additional file 3: Figure S1A,B), which possesses lower Beclin-1 expression compared with other iOvCa lines (Fig. 1f). Thus, representative ovarian cancer cell lines of low, intermediate, and high endogenous Beclin-1 expression are able to robustly induce autophagy.

### Efficient Beclin-1 knockdown does not block autophagy induction in ovarian cancer cell lines

We have demonstrated that ovarian tumors and cell lines maintain Beclin-1 protein expression, and that *in vitro* cultures of these cells retain the capacity to upregulate autophagy. To directly test the contribution of Beclin-1 to autophagy, we used RNA interference to knock down its expression.

**Fig. 4:**
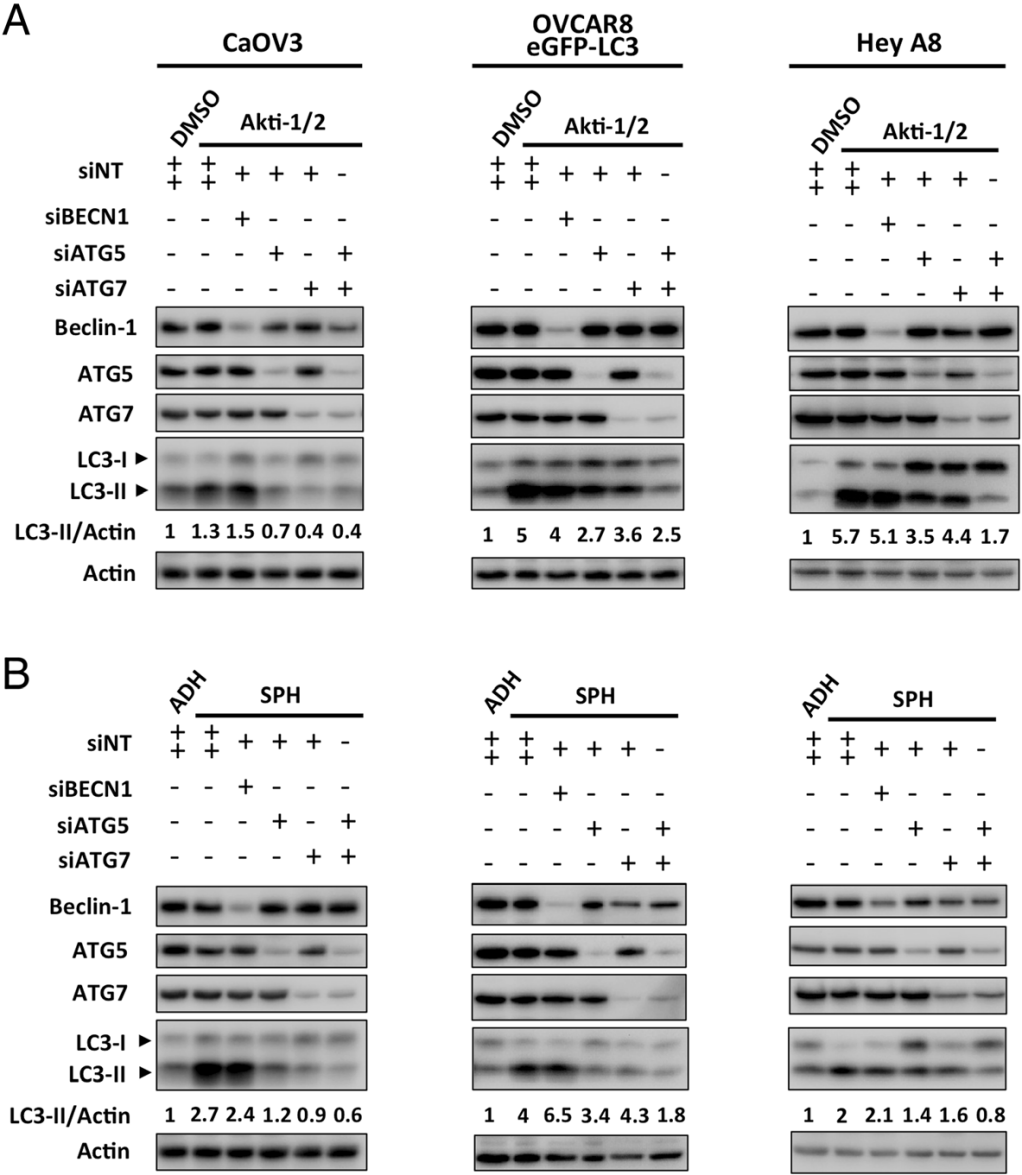
Beclin-1 knockdown does not block autophagy induction in ovarian cancer cell lines with low, intermediate, and high levels of Beclin-1 expression. CaOV3, OVCAR8 eGFP-LC3, and HeyA8 cells were each transfected with control siRNA (siNT) or siRNA targeting *BECN1*, *ATG5*, *ATG7*, or *ATG5* + *7* and cells seeded to adherent or non-adherent culture. **a** Adherent cells were allowed to attach overnight, treated with DMSO or Akti-1/2 (5 μM) the next day, and harvested 24 h later to generate protein lysates. Immunoblots were performed for indicated proteins. **b** Spheroids (along with a parallel adherent culture transfected with control siRNA) were harvested 24 h after seeding to non-adherent culture. Protein lysates were generated and immunoblot performed for indicated proteins. Immunoblots depicted are representative of duplicate experiments

Rather than a single siRNA targeting *BECN1*, we chose to use a ‘SMARTpool’ of siRNAs in an attempt to ensure efficient knockdown. Transfection with siRNAs against *BECN1* efficiently reduced its protein level in CaOV3, OVCAR8, and HeyA8 cells. Expression of the canonical autophagy regulatory proteins ATG5 and ATG7 was also efficiently reduced using RNA interference (Fig. 4). In adherent cells, knockdown significantly reduced levels of Beclin-1 protein, but this had no effect on Akti-1/2-mediated autophagy upregulation as LC3-II accumulation was not correspondingly blocked. This phenomenon was observed in all lines tested: CaOV3, OVCAR8, HeyA8 (Fig. 4a) as well as SKOV3 (Additional file 4: Figure S2A). Replicate immunoblots were quantified and statistically analyzed, demonstrating significant Beclin-1 knockdown (*p* < 0.001) but unchanged LC3-II (Additional file 5: Figure S3A). In contrast, knockdown of ATG5, ATG7, or their combination, effectively blocked LC3-II accumulation as expected (Fig. 4a). In SKOV3 cells, only knockdown of ATG7 effectively blocked LC3-II accumulation (Additional file 4: Figure S2A).

Similar results were seen during spheroid formation, whereby Beclin-1 knockdown failed to block autophagy upregulation. This was observed in CaOV3, OVCAR8, and HeyA8 (Fig. 4b) as well as SKOV3 cells (Additional file 4: Figure S2B). Replicate immunoblots were quantified and analyzed, confirming significant Beclin-1 knockdown (*p* < 0.05) but unchanged LC3-II (Additional file 5: Figure S3A). Nonetheless, spheroid-induced autophagy was effectively blocked upon knockdown of ATG5, ATG7, or both, as evidenced by suppression of LC3-II (Fig. 4b). In SKOV3 cells, only knockdown of ATG7 was able to suppress LC3-II (Additional file 4: Figure S2B).

**Fig. 5:**
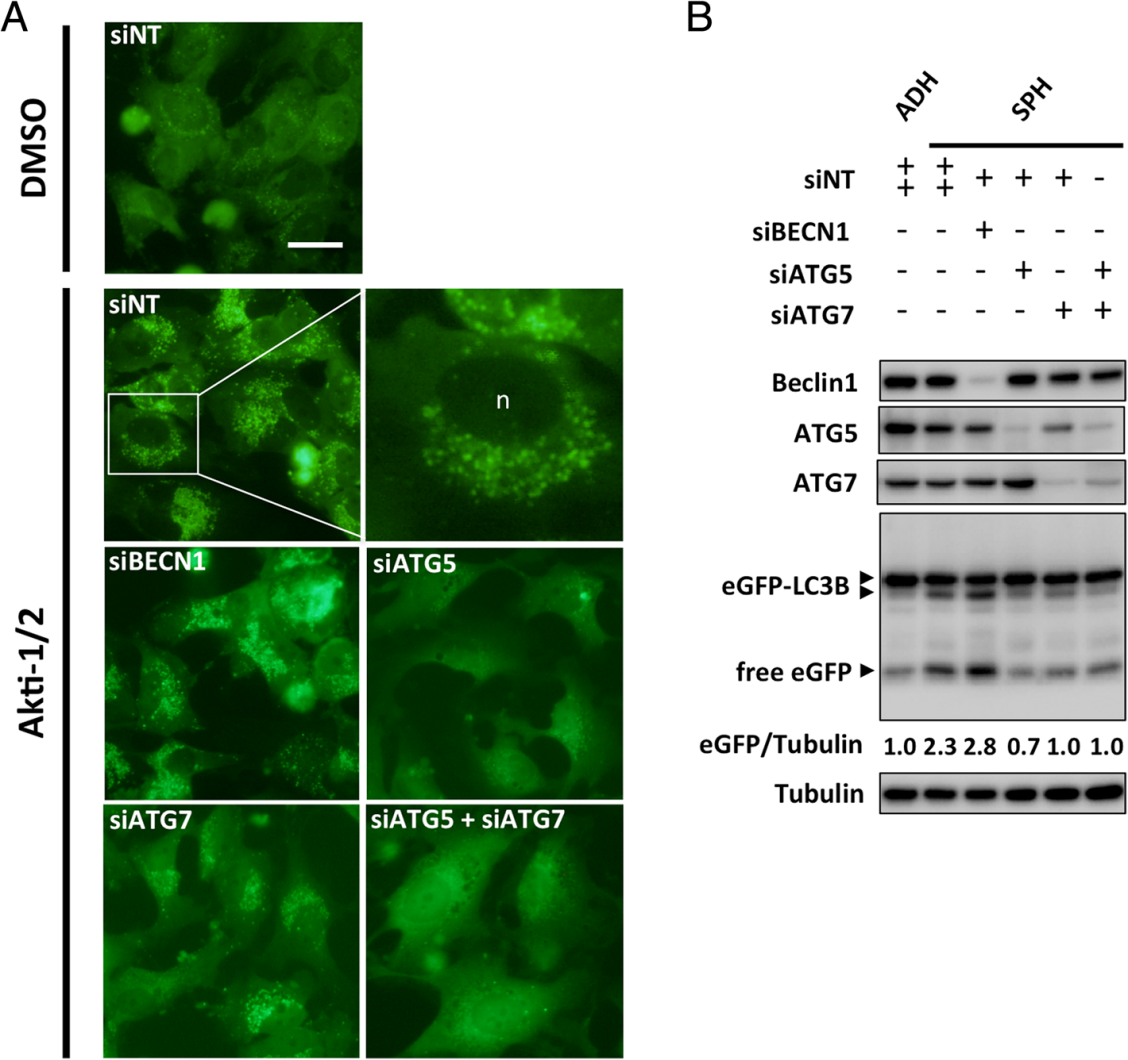
Autophagy induction in OVCAR8-eGFP-LC3B cells is unaffected by Beclin-1 knockdown. OVCAR8-eGFP-LC3 cells were transfected with control siRNA (siNT) or siRNA targeting BECN1, ATG5, ATG7, or ATG5 + 7 and seeded to adherent or non-adherent culture. **a** Adherent cells were allowed to attach overnight and treated with DMSO or Akti-1/2 (5 μM) the next day. Images were captured 24 h post-treatment using an Olympus IX70 inverted microscope and ImagePro software. A portion of the siBECN1 image was expanded to clearly show an abundance of fluorescent puncta around the nucleus (n). Scale bar: 50 μm. **b** Transfected cells seeded to non-adherent culture were harvested 24 h following seeding, protein lysates generated, and immunoblot performed for indicated proteins. An anti-GFP antibody was used to detect fused and free eGFP protein. Images and immunoblot are representative of duplicate experiments

To extend these analyses and provide an alternate method for monitoring autophagy, OVCAR8 cells were generated to stably express an eGFP-labeled LC3B protein. Upon autophagy induction, the cytoplasmic distribution of this fusion protein shifts from a diffuse to punctate appearance as it is incorporated into forming autophagosomes, thus providing visual confirmation that the process is underway [35]. OVCAR8-eGFP-LC3 cells were treated with Akti-1/2 to induce autophagy, prompting a clear shift from diffuse to punctate fluorescence compared to cells treated with vehicle control (Fig. 5a). Knockdown of ATG5, ATG7, or their combination under the same autophagy-inducing conditions resulted in diffuse fluorescence throughout the cytoplasm indicating inhibition of autophagy. In contrast, Beclin-1 knockdown had no effect on puncta formation in the presence of Akti-1/2 (Fig. 5a).

Expression of eGFP-tagged LC3B can also be used in a functional readout of autophagic degradation to further confirm the relocalization of the fluorescent LC3B signal. During autophagy, proteolytic enzymes destroy processed LC3 (LC3-II) that is inserted into the inner autophagic membrane. However, the relative stability of eGFP protects it from a similar fate, and therefore, the abundance of solitary eGFP on immunoblot can be interpreted as a functional readout of autophagic degradation [35]. We utilized this assay in spheroids since their three-dimensional architecture precludes routine live-cell fluorescence microscopy. OVCAR8-eGFP-LC3 cells were cultured to form spheroids, causing an increase in free eGFP and thus implying induction of autophagy (Fig. 5b). This elevated level of free eGFP was undiminished in cells subjected to Beclin-1 knockdown but was blocked by ATG5 and/or ATG7 knockdown (Fig. 5b).

**Fig. 6:**
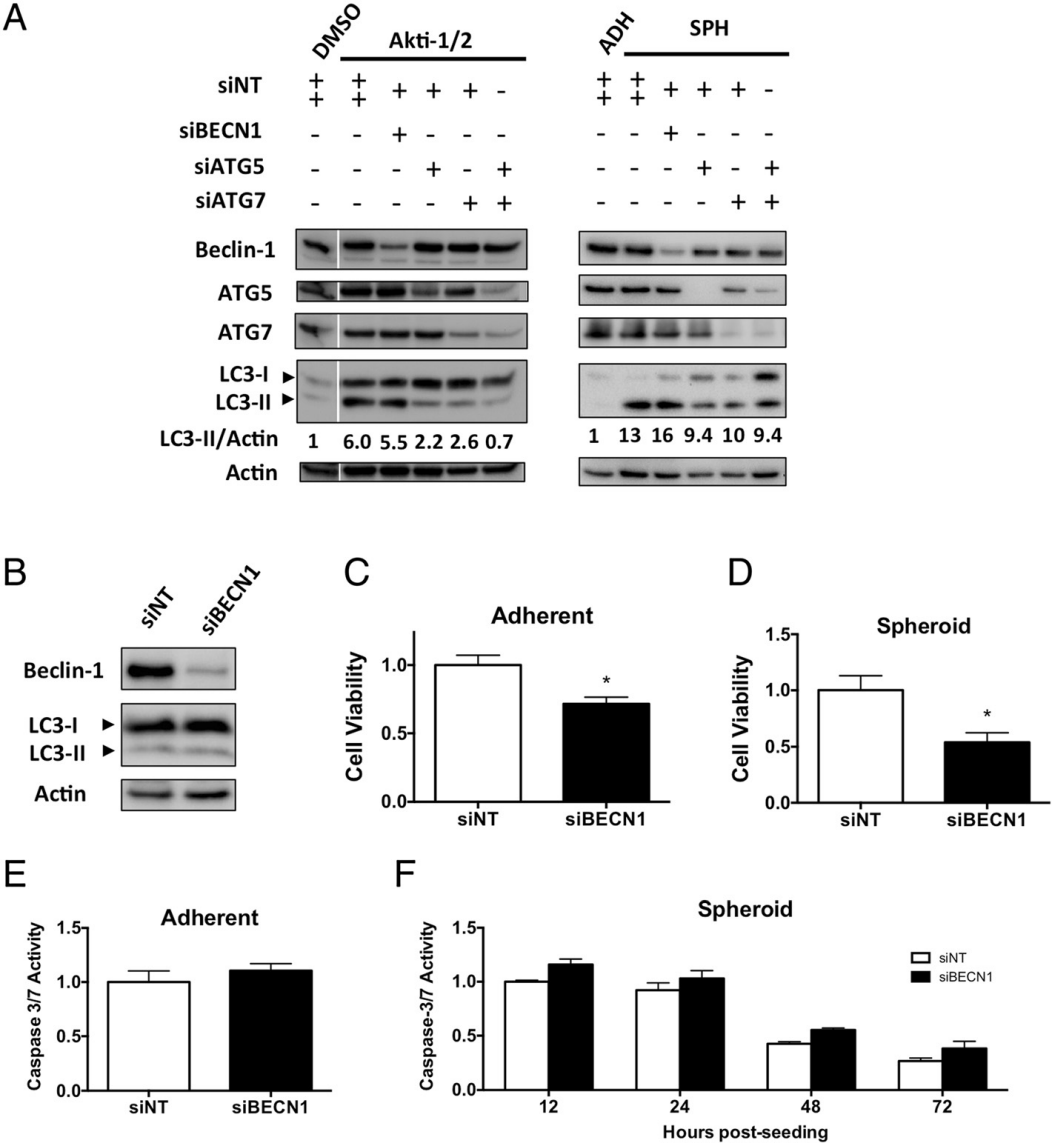
Beclin-1 knockdown reduces cell viability without altering autophagy or apoptosis. **a** iOvCa147-E2 cells were transfected with control siRNA (siNT) or siRNA targeting *BECN1*, *ATG5*, *ATG7*, or *ATG5* + *7* and cells seeded to adherent or non-adherent culture. Adherent cells were allowed to attach overnight, treated with DMSO or Akti-1/2 (5 μM) the next day, and harvested 24 h later to generate protein lysates. Spheroids (along with a parallel adherent culture transfected with control siRNA) were harvested 24 h after seeding to non-adherent culture. Immunoblots were performed (n = 3 repeated experiments) with a representative blot depicted (unrelated, intervening lanes cropped). **b** iOvCa147-E2 cells were transfected with control siRNA (siNT) or siRNA targeting BECN1 and immunoblot performed to verify Beclin-1 knockdown in adherent cells (96 h post-transfection). **c** Viable cells were also counted at this time using Trypan Blue exclusion and normalized to control. Bars: Mean ± SEM (n = 3, *p < 0.05). **d** In spheroid cultures, viability was assessed using the CellTiter-Glo assay (7 days post-transfection and 72 h post-seeding to non-adherent culture). Cell viability data are normalized to controls. Bars: Mean ± SEM (n = 3, *p < 0.05). **e** Apoptosis was quantified using a Caspase 3/7 activity assay in adherent cultures at 96 h post-transfection. **f** Transfected cells were then seeded to non-adherent culture and apoptosis quantified in spheroids at indicated time points. Caspase 3/7 activity data were normalized to controls. Bars: Mean ± SEM (n = 3)

Our findings were confirmed in the ascites-derived, early-passage cell line iOvCa147-E2, which was also transfected with siRNAs against *BECN1*, *ATG5*, and *ATG7*. As before, significant Beclin-1 knockdown (*p* < 0.05) could not disrupt autophagy induced by Akti-1/2 (Fig. 6a, Additional file 5: Figure S3C) or spheroid formation (Fig. 6a, Additional file 5: Figure S3D). However, autophagy could be blocked by knockdown of ATG5 and ATG7, or their combination (Fig. 6a).

Finally, we wished to assess the contribution of Beclin-1 to basal autophagy (i.e., not induced by Akti-1/2 or spheroid formation). Again, Beclin-1 knockdown yielded significantly reduced protein levels (*p* < 0.05), yet no change in LC3-II in SKOV3 [Additional file 4: Figure S2B (‘A’ lanes) and Additional file 6: Figure S4B], HeyA8 (Additional file 6: Figure S4A, C), and iOvCa147-E2 (Additional file 6: Figure S4A, D) cells; whereas basal levels of LC3-II were decreased by knockdown of ATG5, ATG7, or both (Additional file 4: Figure S2, Additional file 6: S4A).

Interestingly, we noted a trend toward slightly increased LC3-II levels upon Beclin-1 knockdown in multiple lines, but this was subtle and of unclear significance. Regardless of this, in no instance did Beclin-1 knockdown yield corresponding decreases in LC3-II, as described above.

These data reveal that the capacity of ovarian cancer cells to undergo autophagy *in vitro* is not tied to their expression of the canonical autophagy regulatory protein Beclin-1. In fact, efficiently knocking down Beclin-1 in cell lines with low, intermediate, or high endogenous expression had no effect on autophagy induction or basal levels of this process.

### Beclin-1 knockdown reduced ovarian cancer cell viability without altering levels of autophagy or apoptosis

Despite a lack of effect on autophagy, we observed that efficient Beclin-1 knockdown in iOvCa147-E2 cells (Fig. 6b) caused significant decreases in cell viability (Fig. 6c,d). In adherent cells counted using Trypan Blue exclusion, a 30 % reduction in viable cell number was observed relative to controls (Fig. 6c). Similarly, spheroid cells exhibited a 50 % reduction in viability as a result of Beclin-1 knockdown (Fig. 6d). Likewise, in HeyA8 cells, Beclin-1 knockdown significantly reduced viability by 30 % in adherent cells and by 15 % in spheroids (Additional file 7: Figure S5). Therefore, our findings in iOvCa147-E2 and HeyA8 cells demonstrate the dispensability of Beclin-1 for autophagy induction, yet suggest a possible role for this this protein in contributing to ovarian cancer cell viability.

Since Beclin-1 is also known to regulate apoptosis independent of its role in autophagy (reviewed in [36]), we postulated that its importance in maintaining iOvCa147-E2 cell viability could involve anti-apoptotic mechanisms. To test this, we performed luminescence-based Caspase 3 & 7 activity assays to assess apoptotic activity upon Beclin-1 knockdown. In both adherent (Fig. 6e) and spheroid cells (Fig. 6f), however, no significant increase in apoptosis induction was observed. This result was confirmed by using a commercially available antibody array specific for apoptosis-related proteins; this approach did not reveal an obvious and significant alteration in apoptotic pathway protein abundance associated with reduction in Beclin-1 levels (Additional file 8: Figure S6).

## Discussion

Beclin-1 has gained notoriety due to its role in mediating autophagy and its role as a tumor suppressor in genetically modified mice. Furthermore, *BECN1* localizes to the long arm of chromosome 17, a region that sees a high frequency of monoallelic loss in high-grade serous ovarian cancer (HGSC). The present study has demonstrated that Beclin-1 protein expression is maintained in spite of prevalent single-copy loss. On further investigation, however, knockdown of Beclin-1 protein did not diminish autophagy induction: both non-pharmacologic and pharmacologic stimuli (spheroid formation and AKT inhibition, respectively) induced autophagy despite significantly decreased Beclin-1 levels, implying a decoupling of Beclin-1 from autophagy regulation. Knockdown did reduce cell viability without inducing apoptosis, suggesting that in HGSC, Beclin-1 may in fact perform important autophagy- and apoptosis-independent functions. This work is the first to describe retained Beclin-1 protein expression in hemizygous tumors and to systematically assess Beclin-1-independent autophagy in ovarian cancer. Our findings move beyond those of prior studies, providing functional data that challenges the accepted understanding of Beclin-1 in autophagy regulation.

Our description of spheroid-associated autophagy also brings together novel concepts pertaining to ovarian cancer metastasis. The concept of dormancy in ovarian cancer spheroids (characterized by cellular quiescence) has previously been demonstrated by our group and others [37, 33, 38]. Separately, the concept of autophagy as an essential feature of dormancy in ovarian tumor xenografts was described in a seminal publication from Bast’s group [25]. Our demonstration of autophagy in spheroids unifies these two concepts for the first time in ovarian cancer, begging the question of whether autophagy may be another critical feature contributing to the dormant phenotype of spheroids. It will be of interest to determine the extent to which autophagy is required for spheroid dormancy and whether autophagy-modulation in the clinical setting could impact disease progression.

A novel finding of this study is Beclin-1 protein expression in 398 HGSCs, despite 76 % of these tumors harboring single-allele loss of *BECN1*. It is in keeping with recent immunohistochemical analyses of Beclin-1 expression that have demonstrated robust staining in ovarian carcinomas [27] greater than that seen in benign tumors or normal ovary [39, 40]. None of these studies, however, evaluated Beclin-1 protein expression in ovarian tumors with respect to gene copy-number as we have done using TCGA datasets.

We initially hypothesized that the maintenance of Beclin-1 expression underlined its importance for autophagy in ovarian tumor cells. Yet surprisingly, we discovered that knockdown of Beclin-1 had no effect on autophagy induction. Such a discovery is not without precedent, since evidence of Beclin-1-independent autophagy has been emerging and is documented in neuronal [41, 42], macrophage [43], and breast cancer cells [44]. Even in ovarian cancer, Beclin-1-independent autophagy has been reported in the context of non-canonical stimuli such as arsenic trioxide [31] and prolactin receptor antagonism [45]. Our study, however, represents the first effort to systematically address Beclin-1-independence in early-passage, ascites-derived ovarian cancer cells as well as established ovarian cancer lines selected for their differing *BECN1* gene copy-number as well as mRNA and protein expression. Moreover, we demonstrate Beclin-1-independent autophagy as a result of both a pharmacologic (AKT inhibition) as well as a novel non-traditional stimulus (spheroid formation). It will be important to demonstrate Beclin-1-independence in an *in vivo* model of metastatic ovarian cancer. Nonetheless, our work represents an important first step, demonstrating this phenomenon in multiple selected cell lines using molecular techniques that are best applied *in vitro*.

The apparent dispensability of Beclin-1 for autophagy induction in ovarian cancer cell lines and its role in contributing to cell viability in HeyA8 and iOvCa147-E2 cells suggests that it has other functions in cancer. Autophagy-unrelated Beclin-1 functions have been suspected since the first mouse models of its disruption were generated. Mice with homozygous *Becn1* disruption (*Becn1*^−/−^) die by embryonic day 7.5 [46], whereas *Atg5*^−/−^ and *Atg7*^−/−^ mice are able to complete embryonic development and survive to birth, eventually succumbing to autophagy insufficiency during the neonatal starvation period and dying within 24 h [47, 48]. Furthermore, the tumor spectrum of *Becn1*^+/−^ mice differs from other mouse models of autophagy disruption: *Becn1*^+/−^ mice develop hepatocellular carcinomas, lung carcinomas, and lymphomas [9] whereas tissue-specific deletion of *Atg7* or mosaic deletion of *Atg5* led to only benign liver adenomas [49] and never the hepatocellular carcinomas seen in *Becn1*^+/−^ mice. This discordance in both knockout phenotype as well as tumor spectrum suggests additional functions of Beclin-1 separate from its role in autophagy. In fact, evidence for its specific autophagy-independent functions continues to emerge (reviewed in [50]). In complex with Class III PI3K, p150 and other co-factors, Beclin-1 functions in Toll-Like Receptor-mediated phagocytosis in macrophages [51], in endocytic degradation of the Epidermal Growth Factor Receptor (EGFR) and in cytokinesis in HeLa cells [52]. Fremont *et al.* also demonstrated a critical role for Beclin-1 in kinetochore assembly and chromosome congression that is not only autophagy-independent, but also independent of the Class III PI3K and p150 core complex [53]. Thus, *in vitro* and *in vivo* data support Beclin-1’s non-exclusivity to autophagy regulation, supporting the idea that its retained expression may fulfill autophagy-independent function(s). It will be important to evaluate precisely how this occurs; first by determining its importance to cancer cell viability, proliferation, and metastasis. These experiments could uncover novel and important oncogenic functions of Beclin-1 that are applicable to other disease sites.

## Conclusions

In conclusion, our findings challenge the accepted relationship between Beclin-1 and autophagy in ovarian cancer and prompt a re-evaluation of the existing model. While we cannot exclude the possibility that autophagy may initially be disrupted by single allele loss of *BECN1* to promote ovarian tumorigenesis, we have clearly shown that Beclin-1 is present in advanced-stage tumors and tumor cells. It is conceivable that initial disruption of Beclin-1 and autophagy forces adaptation of the autophagic machinery to no longer depend upon Beclin-1, yet a role for this protein in maintaining tumor cell viability may select for its continued expression. Further study is needed to not only elucidate the complex and context-dependent roles of Beclin-1 in ovarian and other tumors, but also to understand the often paradoxical nature of autophagy in cancer biology.

## Additional files

Additional file 1: Table S1. Summary of ovarian cancer patient clinical data. (PDF 60 kb) Additional file 2: Table S2. Summary of TCGA ovarian cancer patient clinical data (provisional dataset). (XLS 71 kb) Additional file 3: Figure S1. Ovarian cancer adherent cells and spheroids undergo autophagic flux. (A) Adherent cultures were subjected to 24h treatment with Akti-1/2 (5μM) ± Chloroquine (CQ 50μM) pulse in the final 4h preceding lysis. (B) Lysates were also obtained from parallel spheroid cultures (24h) ± CQ pulse (final 4h). Immunoblots were performed for indicated proteins and LC3-II expression quantified relative to Actin (n = 2 repeated experiments). (PNG 488 kb) Additional file 4: Figure S2. Beclin-1 knockdown fails to block autophagy induction in adherent and spheroid cultures of SKOV3 cells. Adherent SKOV3 cells were transfected with control siRNA (siNT) or siRNA targeting *BECN1*, *ATG5*, or *ATG7*. (A) Cells were seeded to adherent culture, allowed to attach overnight, treated with DMSO or Akti-1/2 (5μM) the next day, and harvested 24h later to generate protein lysates. (B) Spheroids (along with a parallel adherent culture transfected with control siRNA) were harvested 24h after seeding to non-adherent culture. Depicted immunoblots are representative of triplicate experiments. (PNG 735 kb) Additional file 5: Figure S3. Beclin-1 knockdown significantly reduces Beclin1 protein but is unable to suppress LC3-II. (A,B) Protein expression quantification data (Beclin-1 and LC3-II, relative to Actin) from CaOV3, OVCAR8, HeyA8, and SKOV3 cells were pooled for adherent (A) and spheroid (B) experiments. Bars: Mean ± SEM (n = 2–3 experiments per cell line, 4 cell lines pooled. Paired *t*-test was performed (NS = no significant difference, **p*<0.05, ****p*<0.001). (C,D) Expression of Beclin-1 and LC3-II was quantified in iOvCa147-E2 adherent (C) and spheroid (D) cells. Bars: Mean ± SEM (n = 3 independent experiments; NS = no significant difference, **p*<0.05, *****p*<0.0001). (PNG 246 kb) Additional file 6: Figure S4. Beclin-1 knockdown fails to suppress basal levels of autophagy in adherent ovarian cancer cells. (A) Adherent HeyA8 and iOvCa147-E2 cells were transfected with control siRNA (siNT) or siRNA targeting *BECN1*, *ATG5*, *ATG7, or ATG5 and ATG7*. Cells were re-seeded to adherent culture and harvested 48h later to generate protein lysates. Depicted immunoblots are representative of triplicate experiments. (B,C,D) Protein expression of Beclin-1 and LC3-II was quantified in unstimulated (B) SKOV3, (C) HeyA8, and (D) iOvCa147-E2 cells. Bars: Mean ± SEM (n = 3 independent experiments per cell line; NS = no significant difference, **p*<0.05, ***p*<0.01). (PNG 393 kb) Additional file 7: Figure S5. Beclin-1 knockdown reduces the viability of HeyA8 adherent and spheroid cells. Adherent HeyA8 cells were transfected with control siRNA (siNT) or siRNA targeting *BECN1*. Cells were seeded to either adherent or spheroid culture, and 72h later, subjected to viability assessment using the CellTiter-Glo assay. Bars: Mean ± SEM (n = 3 independent experiments; **p*<0.05, ***p*<0.01). (PNG 127 kb) Additional file 8: Figure S6. Apoptosis-related protein expression is largely unaltered in adherent and spheroid cells upon Beclin-1 knockdown. Adherent iOvCa147-E2 cells were transfected with control siRNA (siNT) or siRNA targeting *BECN1*. Cells were seeded to either adherent or spheroid culture, and 24h later, protein lysates were harvested and apoptosis arrays processed as per manufacturer’s protocol. Bars: Mean ± SD of duplicate array dots. (PNG 208 kb)

## Abbreviations

BECN1: Beclin-1
TCGA: The Cancer Genome Atlas
HGSC: high-grade serous ovarian cancer
mTORC1: mechanistic target of Rapamycin complex 1
atg: autophagy-related proteins
PI3K C3: phosphoinositide-3-kinase, class 3
GISTIC: Genomic Identification of Significant Targets
RPPA: reverse-phase protein array
CCLE: The Cancer Cell Line Encyclopedia
aCGH: array comparative genomic hybridization
EOC: Epithelial ovarian cancer
TEM: transmission electron microscopy
APL: autophagolysosomes
TCPA: The Cancer Proteome Atlas
CQ: Chloroquine.
