# Supplementary material for: Beclin-1 expression is retained in high-grade serous ovarian cancer yet is not essential for autophagy induction in vitro
Source: J Ovarian Res. 2015 Aug 4;8:52. doi: 10.1186/s13048-015-0182-y (PMC4524172; doi:10.1186/s13048-015-0182-y)
Supplement: Additional file 1: Table S1. — Summary of ovarian cancer patient clinical data. (PDF 60 kb) [file 13048_2015_182_MOESM1_ESM.pdf]

Supplementary Table 1: Summary of ovarian cancer patient clinical data

Identifier	Age	Primary Tumor Histological Subtype	Grade	Stage
EOC27	77	Serous adenocarcinoma	High	III
EOC30	81	Serous adenocarcinoma	High	IIIC
EOC31	46	Serous adenocarcinoma	High	IIIC
EOC33	79	Serous adenocarcinoma	High	IIIC
EOC35	81	Serous adenocarcinoma	High	IIIC
EOC39	66	Serous adenocarcinoma	High	IIIC
EOC59	47	Serous adenocarcinoma	High	IV
EOC65	67	Serous adenocarcinoma	High	IIIC
EOC67	51	Serous adenocarcinoma	High	IIIC
EOC81	69	Ovarian mucinous borderline tumor	n.a.	IIB
EOC87	47	Serous adenocarcinoma	High	IV
EOC89	56	Serous and clear cell differentiation	High	IV
EOC98	51	Serous adenocarcinoma	High	IIIC
EOC100	65	Serous (>90%) and endometrioid (<10%) adenocarcinoma	High	IIIA
EOC118	57	Serous adenocarcinoma	High	IIIC
EOC122	56	Serous carcinoma	High	IIIC
EOC129	74	Serous carcinoma	High	IIIC
EOC136	42	Serous ovarian carcinoma	High	IV
EOC155	66	Serous adenocarcinoma	High	IIIC
EOC161	67	Serous carcinoma	High	IIIC
EOC166	64	Serous adenocarcinoma	High	IIIC
EOC168ǂ	77	Serous (70%) and clear cell (30%) adenocarcinoma	High	IIIC
EOC171	65	Serous carcinoma	High	IIIC
iOvCa147	43	Serous (70%) and clear cell (30%) adenocarcinoma	High	IIC
iOvCa168	77	Serous (70%) and clear cell (30%) adenocarcinoma	High	IIIC
iOvCa170ǂ	77	Serous (70%) and clear cell (30%) adenocarcinoma	High	IIIC
iOvCa182	58	Serous adenocarcinoma	High	IIIB
iOvCa185	78	Carcinosarcoma with serous, endometrioid, and clear cell differentiation	High	IV
iOvCa198	65	Serous adenocarcinoma	High	IIIB
iOvCa201	76	Serous adenocarcinoma	High	IIC
iOvCa205	73	Serous adenocarcinoma	High	IIB

n.a., not available
