# Supplementary figures and images for: Beclin-1 expression is retained in high-grade serous ovarian cancer yet is not essential for autophagy induction in vitro
Source: J Ovarian Res. 2015 Aug 4;8:52. doi: 10.1186/s13048-015-0182-y (PMC4524172; doi:10.1186/s13048-015-0182-y)

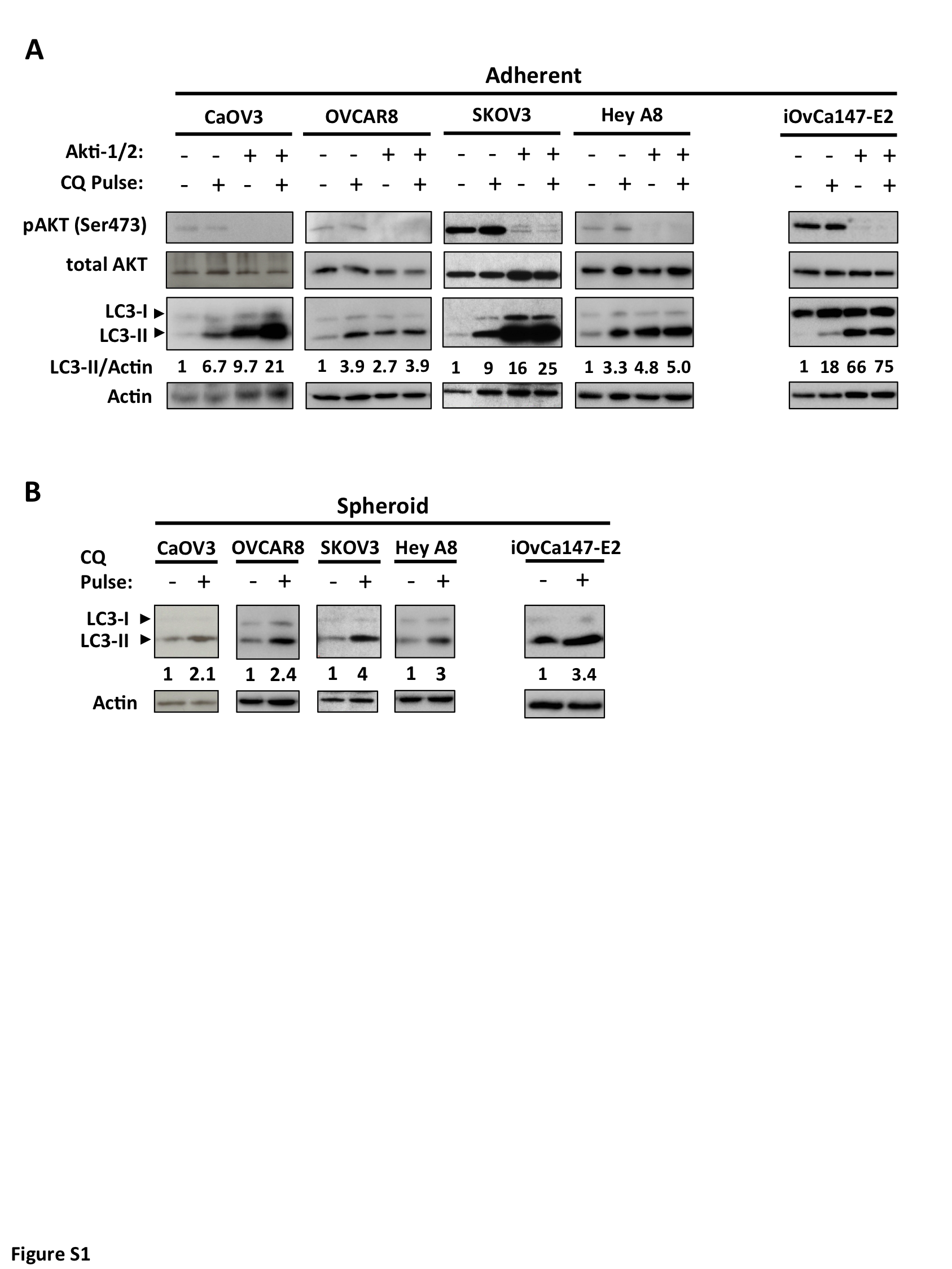

Supplement: Additional file 3: Figure S1. — Ovarian cancer adherent cells and spheroids undergo autophagic flux. (A) Adherent cultures were subjected to 24h treatment with Akti-1/2 (5μM) ± Chloroquine (CQ 50μM) pulse in the final 4h preceding lysis. (B) Lysates were also obtained from parallel spheroid cultures (24h) ± CQ pulse (final 4h). Immunoblots were performed for indicated proteins and LC3-II expression quantified relative to Actin (n = 2 repeated experiments). (PNG 488 kb) [file 13048_2015_182_MOESM3_ESM.png]

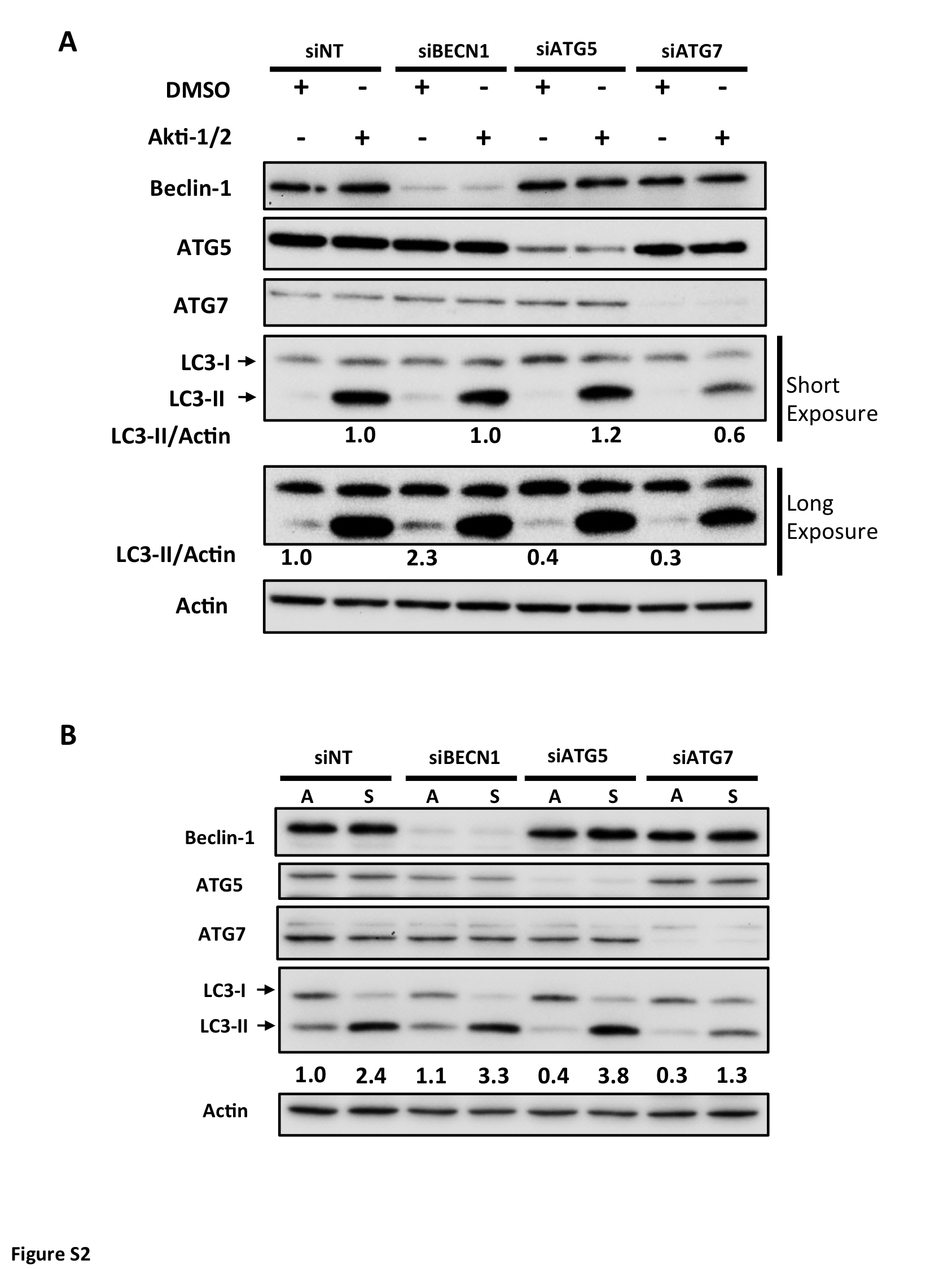

Supplement: Additional file 4: Figure S2. — Beclin-1 knockdown fails to block autophagy induction in adherent and spheroid cultures of SKOV3 cells. Adherent SKOV3 cells were transfected with control siRNA (siNT) or siRNA targeting BECN1, ATG5, or ATG7. (A) Cells were seeded to adherent culture, allowed to attach overnight, treated with DMSO or Akti-1/2 (5μM) the next day, and harvested 24h later to generate protein lysates. (B) Spheroids (along with a parallel adherent culture transfected with control siRNA) were harvested 24h after seeding to non-adherent culture. Depicted immunoblots are representative of triplicate experiments. (PNG 735 kb) [file 13048_2015_182_MOESM4_ESM.png]

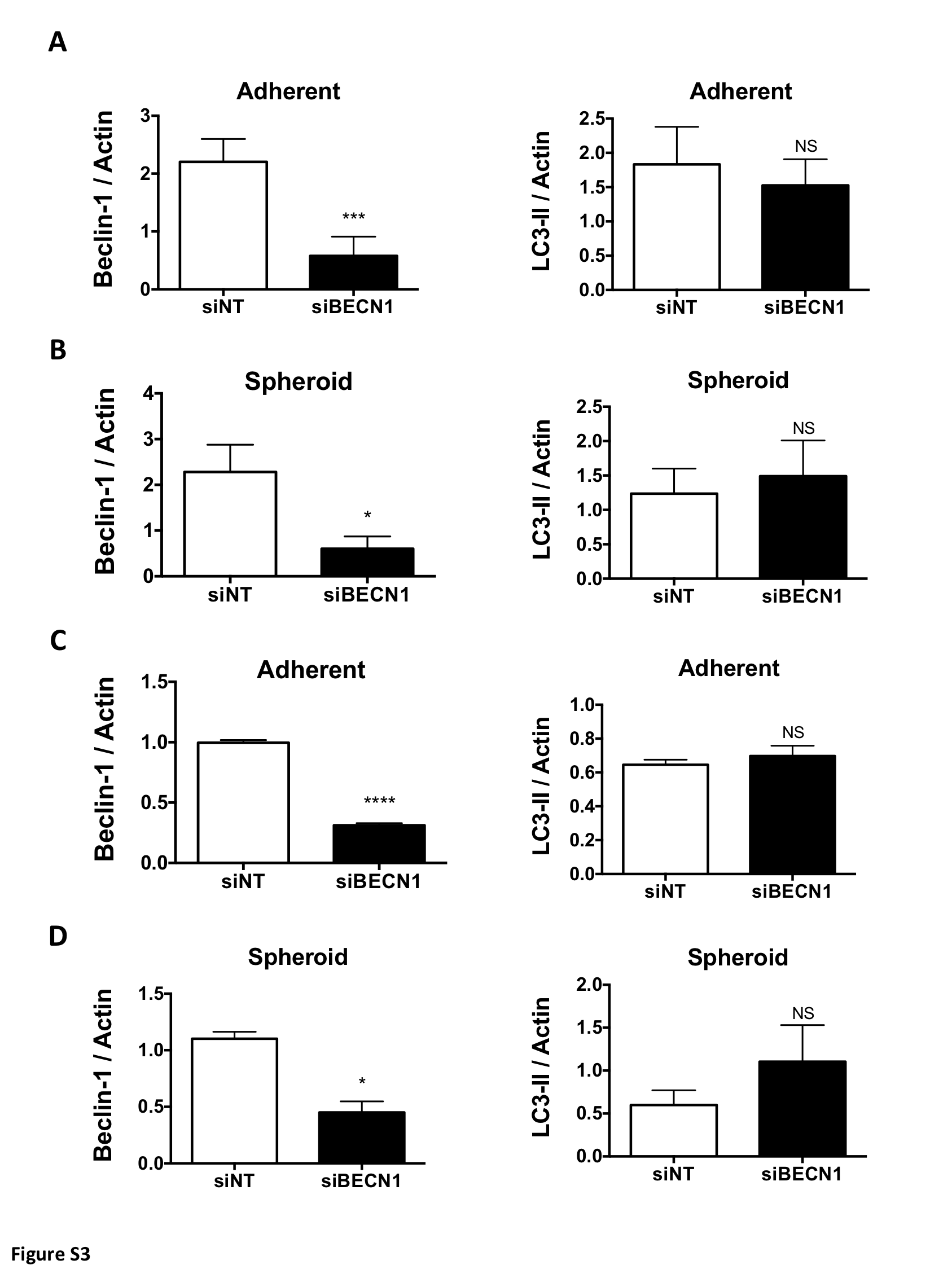

Supplement: Additional file 5: Figure S3. — Beclin-1 knockdown significantly reduces Beclin1 protein but is unable to suppress LC3-II. (A,B) Protein expression quantification data (Beclin-1 and LC3-II, relative to Actin) from CaOV3, OVCAR8, HeyA8, and SKOV3 cells were pooled for adherent (A) and spheroid (B) experiments. Bars: Mean ± SEM (n = 2–3 experiments per cell line, 4 cell lines pooled. Paired t-test was performed (NS = no significant difference, *p<0.05, ***p<0.001). (C,D) Expression of Beclin-1 and LC3-II was quantified in iOvCa147-E2 adherent (C) and spheroid (D) cells. Bars: Mean ± SEM (n = 3 independent experiments; NS = no significant difference, *p<0.05, ****p<0.0001). (PNG 246 kb) [file 13048_2015_182_MOESM5_ESM.png]

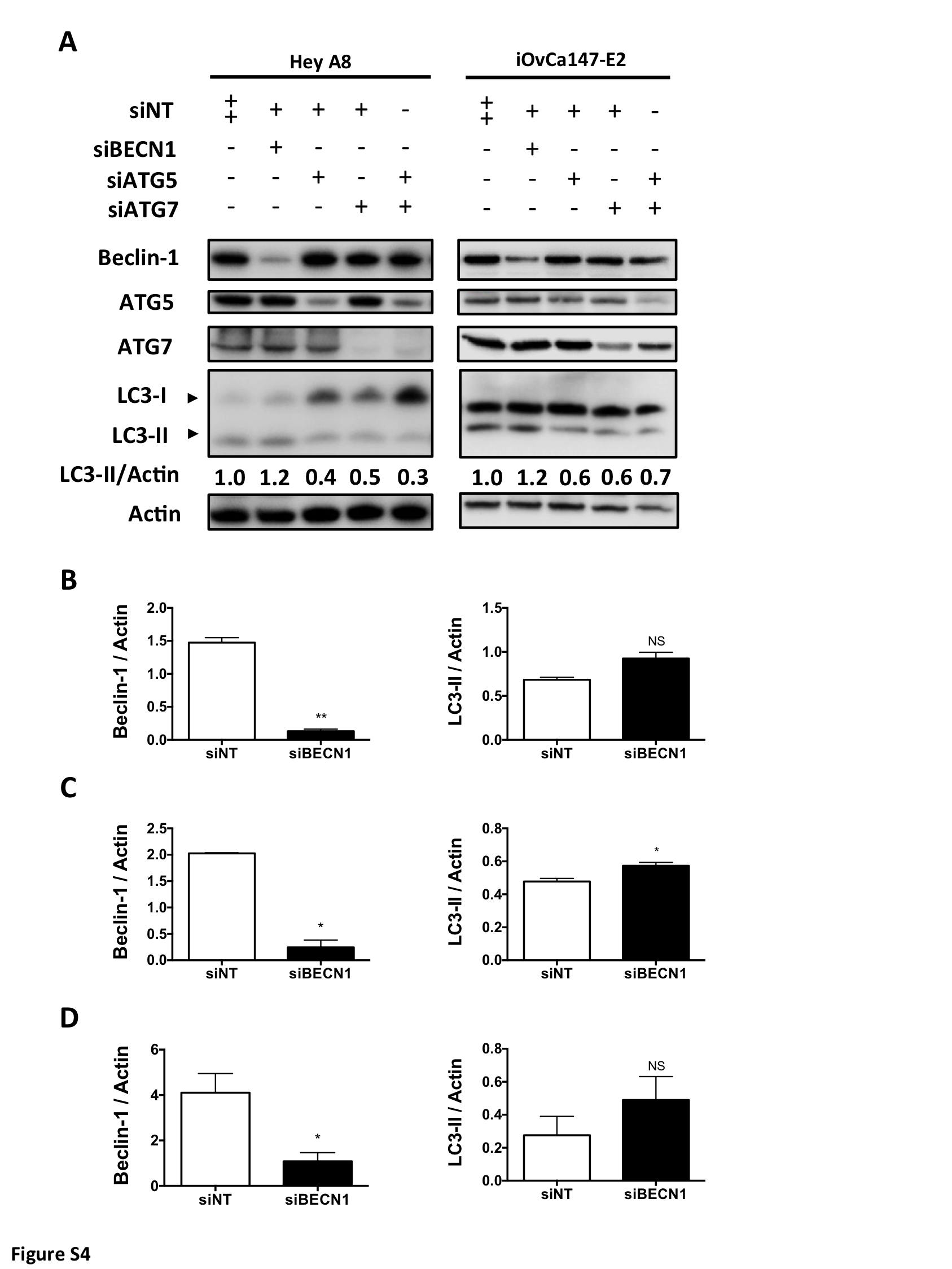

Supplement: Additional file 6: Figure S4. — Beclin-1 knockdown fails to suppress basal levels of autophagy in adherent ovarian cancer cells. (A) Adherent HeyA8 and iOvCa147-E2 cells were transfected with control siRNA (siNT) or siRNA targeting BECN1, ATG5, ATG7, or ATG5 and ATG7. Cells were re-seeded to adherent culture and harvested 48h later to generate protein lysates. Depicted immunoblots are representative of triplicate experiments. (B,C,D) Protein expression of Beclin-1 and LC3-II was quantified in unstimulated (B) SKOV3, (C) HeyA8, and (D) iOvCa147-E2 cells. Bars: Mean ± SEM (n = 3 independent experiments per cell line; NS = no significant difference, *p<0.05, **p<0.01). (PNG 393 kb) [file 13048_2015_182_MOESM6_ESM.png]

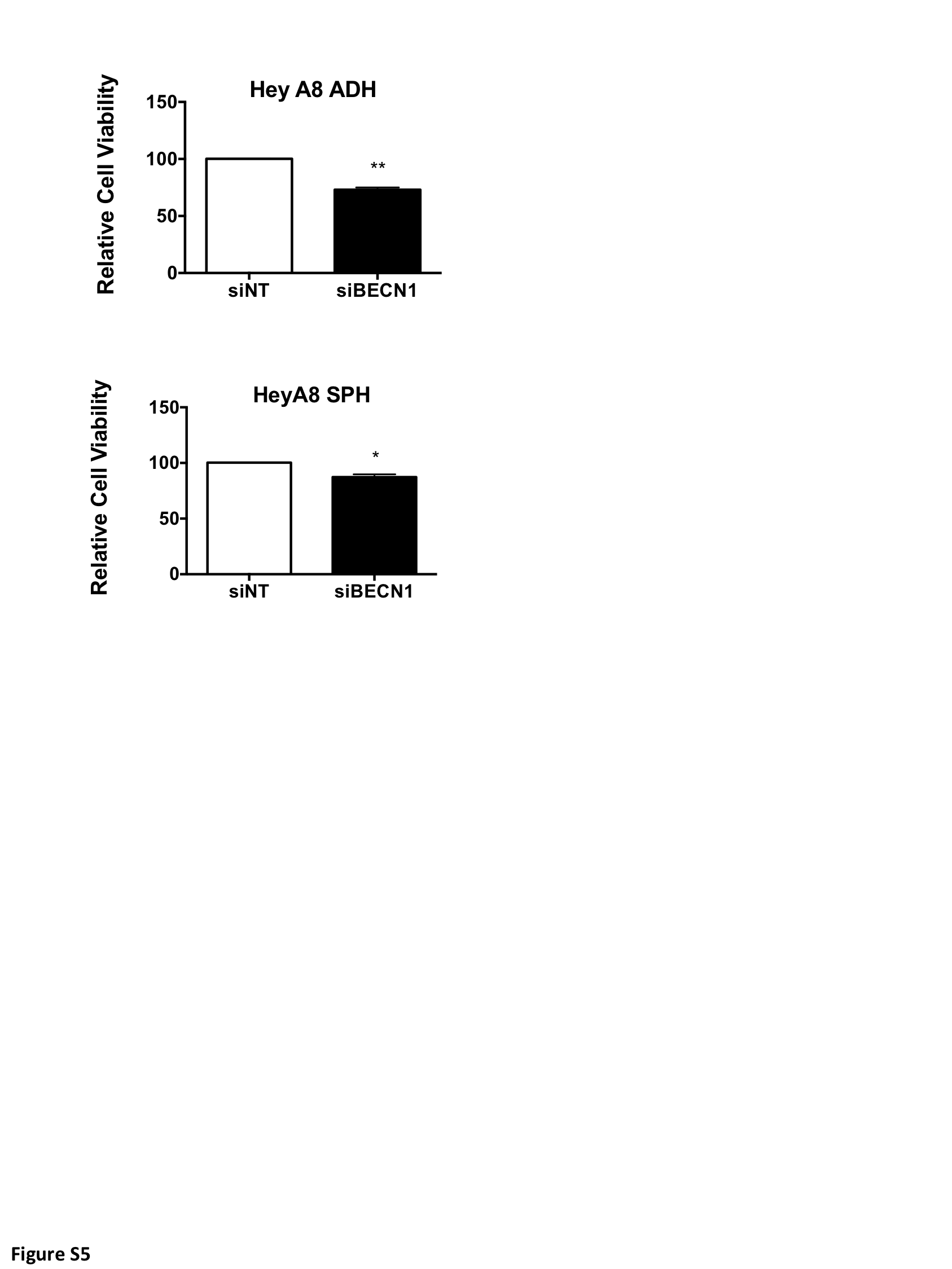

Supplement: Additional file 7: Figure S5. — Beclin-1 knockdown reduces the viability of HeyA8 adherent and spheroid cells. Adherent HeyA8 cells were transfected with control siRNA (siNT) or siRNA targeting BECN1. Cells were seeded to either adherent or spheroid culture, and 72h later, subjected to viability assessment using the CellTiter-Glo assay. Bars: Mean ± SEM (n = 3 independent experiments; *p<0.05, **p<0.01). (PNG 127 kb) [file 13048_2015_182_MOESM7_ESM.png]

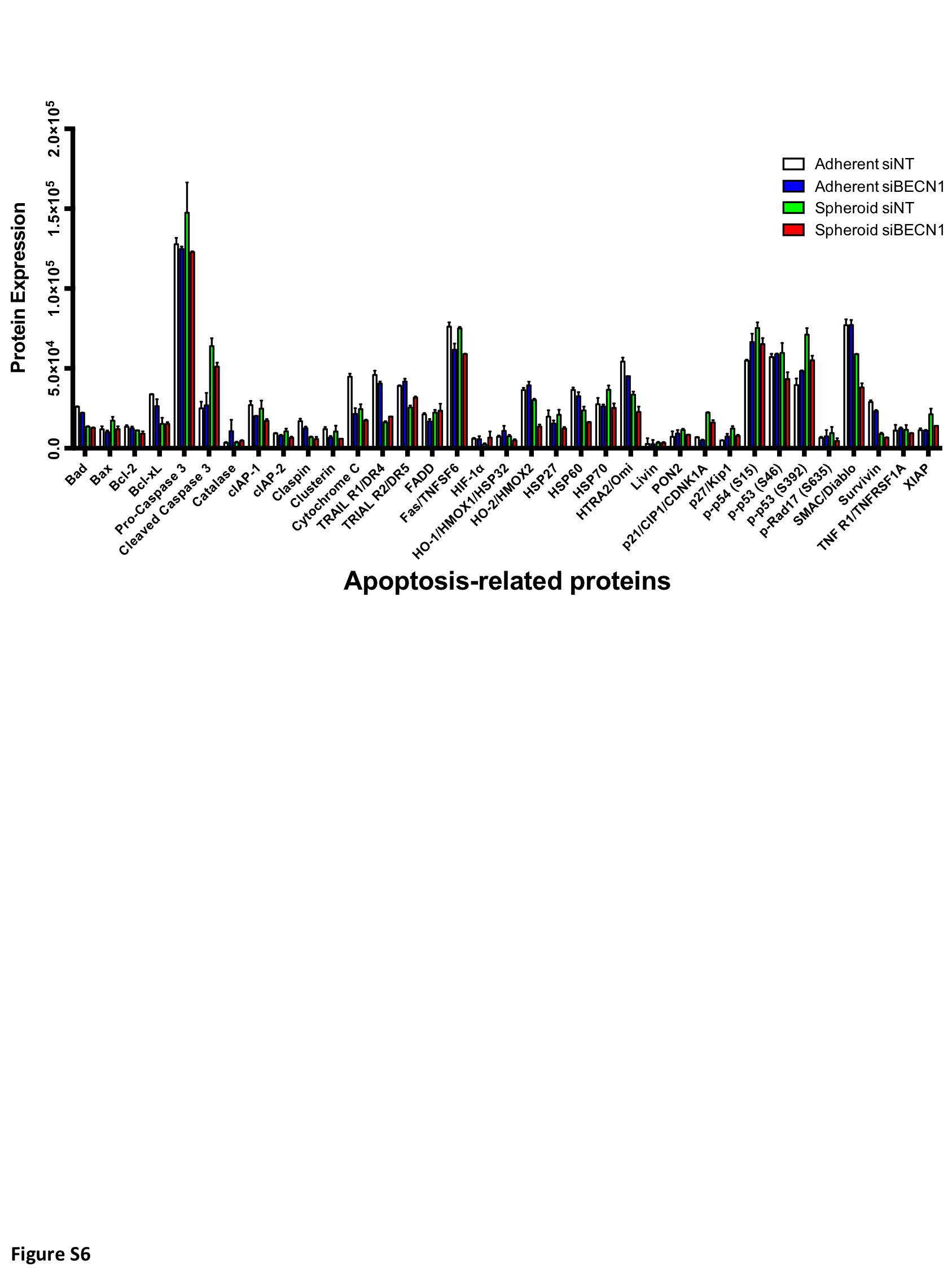

Supplement: Additional file 8: Figure S6. — Apoptosis-related protein expression is largely unaltered in adherent and spheroid cells upon Beclin-1 knockdown. Adherent iOvCa147-E2 cells were transfected with control siRNA (siNT) or siRNA targeting BECN1. Cells were seeded to either adherent or spheroid culture, and 24h later, protein lysates were harvested and apoptosis arrays processed as per manufacturer’s protocol. Bars: Mean ± SD of duplicate array dots. (PNG 208 kb) [file 13048_2015_182_MOESM8_ESM.png]
